# The local nature of incompressibility of quantum Hall effect

**DOI:** 10.1038/ncomms14082

**Published:** 2017-01-10

**Authors:** E. M. Kendirlik, S. Sirt, S. B. Kalkan, N. Ofek, V. Umansky, A. Siddiki

**Affiliations:** 1 Faculty of Science, Department of Physics, Istanbul University, Vezneciler, Istanbul 34134, Turkey; 2 Department of Physics, Science and Letters Faculty, Mimar Sinan Fine Arts University, Sisli, Istanbul 34380, Turkey; 3 Department of Applied Physics, Yale University, 217 Prospect Street New Haven, Connecticut 06511-8499, USA; 4 Braun Center for Submicron Research, Department of Condensed Matter Physics, Weizmann Institute of Science, Rehovot 76100, Israel

## Abstract

Since the experimental realization of the integer quantum Hall effect in a two-dimensional electron system, the interrelation between the conductance quantization and the topological properties of the system has been investigated. Assuming that the two-dimensional electron system is described by a Bloch Hamiltonian, system is insulating in the bulk of sample throughout the quantum Hall plateau due to a magnetic field induced energy gap. Meanwhile, the system is conducting at the edges resembling a 2+1 dimensional topological insulator without time-reversal symmetry. Here, by our magneto-transport measurements performed on GaAs/AlGaAs high purity Hall bars with two inner contacts we show that incompressible strips formed at the edges result in Hall quantization, even if the bulk is compressible. Consequently, the relationship between the quantum Hall effect and topological bulk insulator breaks for specific field intervals within the plateaus. The measurement of conducting bulk, strongly challenges all existing single-particle theories.

## Abstract (web summary)

Edge current quantization in the integer quantum Hall effect is understood to arise due to noninteracting electrons circulating an incompressible insulating bulk. Here, the authors evidence compressible metal-like bulk behaviour in GaAs/AlGaAs Hall bars consistent with electronic interactions.

The mutual relation between the integer quantized Hall effect^1^ (IQHE) and topology is intensively investigated during the last decades theoretically^2^,^3^,^4^,^5^. Salient features of the IQHE are the precise Hall conductance measured as integer multiples of the conductance quanta *e*^2^/*h* (*e* is the elementary charge and *h* is the Planck constant) accompanied by zero longitudinal resistance at certain magnetic field intervals. The robustness of the IQHE independent of material properties points to a universal origin, which has been claimed to be the topology of the system^3^ and is based on the key argument: whenever the Hall conductance is quantized, the bulk of the two-dimensional electron system (2DES) is incompressible^4^. For the IQHE, the incompressible state is a direct consequence of the quantizing magnetic field *B* localizing the electrons when the Fermi energy *E*_F_ falls in a Landau gap. Hence, due to the lack of available states the bulk is insulating, while the number of Landau levels below *E*_F_ determines the filling factor *ν*. In the case of the fractional quantized Hall effect the energy gap emanates from many-body interactions, based on exchange and correlation effects, and *ν* takes particular fractional numbers^5^,^6^. Quite generally, the bulk region is assumed to be incompressible and provides an insulating scattering free region between the probe contacts.

The rationale behind the relation between the incompressible topological bulk insulator and the conductance quantization stems from the mathematical analogy between the Landau Hamiltonian and the Bloch Hamiltonian of a periodic system, which can be well understood physically in terms of the Berry phase, known as Laughlins *Gedankenexperiment*^5^. In the standard picture, the charge transport along the edges is described by single particle, that is, non-interacting, edge states which are formed due to the level bending imposed by hard wall boundary conditions. The edge states carry a non-local and dissipationless current^7^. Assuming periodic boundaries, the Hall conductance *σ*_*xy*_ is related to a topological invariant, namely the Chern number, which can be elegantly proven using the Kubo formalism. In the case of the IQHE with *σ*_*xy*_=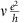, *ν* is the Chern number^8^. Adding two inner contacts to the bulk of the system modifies its topology. Hence, the conductance quantization should be affected, unless the bulk insulating state remains unchanged. To probe whether the bulk remains incompressible one can impose an external AC current excitation between the inner contacts and measure the potential difference as a function of magnetic field *B*. Different from typical Hall experiments, using inner contacts the impedance *Z* measured here not only contains the Ohmic resistance *R* but also a capacitance *C*, |*Z*|=|*R*+*i*/(2*πfC*)|, with *f* being the frequency of the AC excitation. The (quantum) capacitance is given by *C*=*e*^2^*D*_T_ (*E*_F_*A*, where *D*_T_(*E*_F_) is the thermodynamic density of states (TDOS) at *E*_F_ and *A* the area of incompressible region. Within the single-particle picture, the bulk is incompressible and *D*_T_ (*E*_F_)=0 throughout the conductance plateau, the capacitance vanishes and impedance diverges to infinity for an ideally pure 2DES at *T*→0.

In contrast to the above single-particle description of the IQHE, it has also been proposed that the direct Coulomb interaction modifies the electronic distribution and the bulk becomes compressible also within the plateau, that is, the bulk behaves like a metal with a high TDOS at *E*_F_ (refs 9, 10, 11). In this situation, the incompressible states (commonly called strips) reside at the edges of the sample, due to a similar level bending of the Landau levels. Under such conditions, one could drive a current between the inner contacts and measure a finite impedance, without disturbing the perfect quantization measured with outer contacts. There have been few attempts in the literature to clarify the incompressibility of the bulk using contacts, however, most of the experiments utilised indirect measurement techniques, such as capacitive coupling, scanning force microscopy or single electron transistors^12^,^13^,^14^, moreover the samples have been either of low mobility (*μ*<10^6^ cm^2^ V^−1^ s^−1^) and/or large in width *W* (>100 μm). We also note that, disorder can result in localized states creating a finite TDOS around *E*_F_, hence insulators can also be compressible^15^,^16^.

Here we investigate directly the bulk transport properties of a topologically modified Hall bar using our dilution fridge equipped with a 20 Tesla super-conducting magnet at low temperatures (≤750 mK). The potential difference between the inner contacts is measured simultaneously with a standard Hall characterization while an AC excitation is imposed. We find that the bulk of the Hall bar is not entirely insulating all along the conductance plateaus. This observation is in perfect agreement with the predictions of the screening theory of the IQHE^17^, however, challenges the single-particle theories, which incontestably assume an insulating bulk in the plateau interval regardless of the magnetic field strength. Our experimental findings suggest a reexamination of the existing theories of the IQHE from a more general topological point of view and suggest further investigations into the relation between topological insulators and the quantized Hall effect. Inspired by the proposal^17^, we will determine the effects of a current between our inner contacts on the Hall conductance when imposing an excitation between them. Complementary information and discussion are given in Supplementary Notes 1–8.

## Results

### Experimental overview

**Figure 1:**
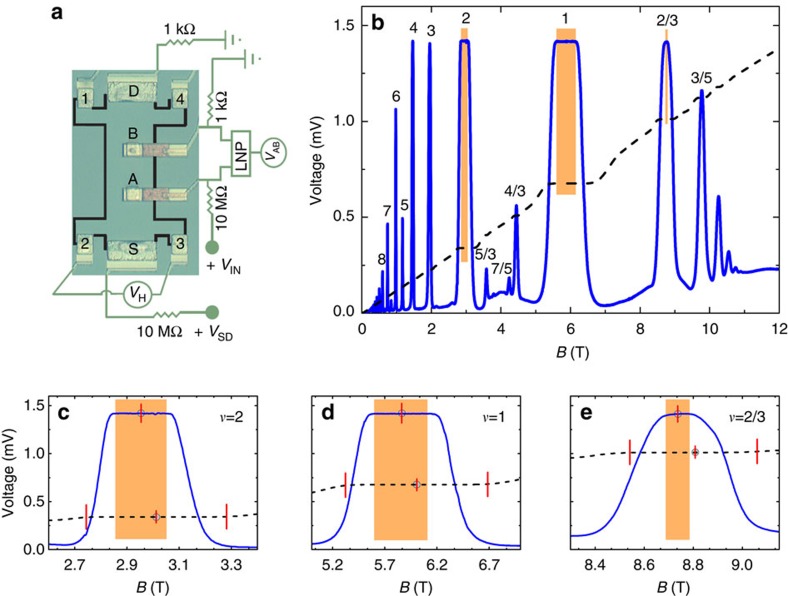
The experimental set-up and measured voltages at base temperature. (**a**) The scanning electron microscope image of the Hall bar (physical edges are highlighted by thick solid lines) defined on high purity GaAs/AlGaAs wafer, together with the measurement set-up. The voltage signal is filtered by a low-noise preamplifier (LNP) and measured by a lock-in amplifier (LIA). On one hand a 26 mV_RMS_ AC excitation is imposed between source-drain contacts (S and D, at 8.54 Hz) *V*_SD_. On the other hand, a 4 mV_RMS_ AC excitation is imposed between inner contacts (A-B, at 11.5 Hz) *V*_IN_, simultaneously. The Hall potential *V*_H_ is measured between contacts 1–4 or 2–3, whereas longitudinal voltage difference is measured between contacts 1–2 or 3–4. (**b**) The Hall potential measured between contacts 1–4 (broken black line) and the potential difference between the inner contacts A-B is measured (*V*_AB_ thick solid blue line) as a function of magnetic field *B*, while imposing AC voltage excitations between contacts A-B and S-D. We also checked that there is no correlation between *V*_AB_ and *V*_SD_ by observing the measured potential differences without one of the excitations. Shaded areas depict the constant *V*_AB_ subintervals observed for *ν*=2,1 and 2/3, which are zoomed in **c**–**e**, respectively. The vertical long-lines indicate the plateau intervals, whereas their relative centres are indicated by vertical short-lines with circles. The constant voltage intervals are determined by a percentage error of 1%.

In this paper, our aim is to show that the bulk of the Hall bar is not incompressible throughout quantum Hall plateaus by direct transport measurements. Our samples are defined on high purity GaAs/AlGaAs wafers, produced at Braun Center for Submicron Research. The Hall bars are 10 *μ*m wide and 40 μm long, defined by chemical etching. Hall contacts are designed like a ‘Lizard' such that they are not effected by the inner contacts. We embedded two inner contacts to the bulk of the Hall bar utilising air bridge technique, each inner contact has ∼1 μm^2^ area and they are 7.5 μm apart from each other. In Fig. 1a we show a scanning electron microscope image of our 10 μm wide Hall bar together with a sketch of the experimental set-up. The 2DES is created in a high mobility (*μ*≈8 × 10^6^ cm^2^ V^−1^ s^−1^, after illumination) GaAs/AlGaAs heterostructure residing 130 nm below the surface. To measure Hall effect we excite the 2DEG by imposing a constant AC voltage between the source and drain contacts at 8.54 Hz by a Lock-in amplifier (LIA, SR 850, with an input impedance of 10 MΩ+25 pF and output impedance of 50 Ω). A 10 MΩ resistance is placed between inner contact A and output of LIA, whereas a 1 kΩ resistance is placed between inner contact B and ground of LIA. We measure Hall voltage (both X and Y components) between contacts 2–3 or 1–4. To check whether the Hall voltage is affected by inner contacts we also reversed source and drain contacts and observed similar features. Similar to Hall voltage measurements, we excite the inner contacts by a lock-in amplifier at 11.5 Hz, followed by a 10 MΩ resistance before source contact S and 1 kΩ resistance after drain contact D. We measure the potential difference between the inner contacts, while a low-noise preamplifier is utilised to filter high-frequency noise (>30 Hz). All the signals go through a room temperature resistance-capacitance (RC) circuit to filter noise above 92 kHz. We used 300 ms time constant and swept the magnetic field by a 0.1 T min^−1^ rate. The measurements are repeated for many thermal cycles and different samples defined on the same wafer. All the results were consistent.

### Measurements at base temperature

Here we present transport measurements from one out of three thermal cycles which were reproduced in two more cycles (also before and after illumination of the sample) considering two different Hall bars defined on different chips to eliminate specific sample properties. We found that all measurements have the same salient features discussed here. Figure 1b depicts the Hall voltage *V*_H_ (dashed) measured with a Lock-in between contacts 1 and 4 as a function of magnetic field *B* (Fig. 1a). We applied a root mean square voltage excitation of 26 mV to source-drain contacts *V*_SD_ over the Hall bar and a 10 MΩ resistance in series (Fig. 1a), which results at a current of *I*=2.6 nA. The cryostats temperature was at *T*_Base_=15 mK. We simultaneously applied an AC voltage *V*_IN_=4 mV (root mean square, corresponding to *I*_IN_=0.4 nA) across the inner contacts A and B, and a 10 MΩ resistance in series (Fig. 1a) and plot the measured *V*_AB_ between A and B as a blue solid line in Fig. 1b. The pronounced local maxima correspond to Hall plateaus at integer or fractional filling factors, as marked in Fig. 1b. For *ν*=2, 1 and 2/3 we observe the absolute maxima of 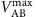
=1.35 meV (highlighted by shaded areas) corresponding *Z*_IN_→∞ within a subinterval of the associated Hall plateau. For these filling factors we provide enlarged views in Fig. 1c–e. We attribute this behaviour to a well established bulk incompressible region as we will explain in the following. Interestingly, the regions in *B* of 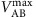 are considerably smaller than the associated Hall plateaus measured with the outer contacts (compare shaded areas to red marks in Fig. 1c–e). For *V*_AB_<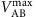 at still constant *V*_H_ the bulk between the inner contacts is compressible while the outer edges of the Hall bar are still protected by incompressible strips. Another interesting feature observed is, the central *B* values of 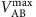 and *V*_H_ do not coincide. In the single-particle pictures, it is assumed that the largest localized state resides at the centre of the plateau. However, here we observe that the largest incompressible region is not at the plateau centre.

In summary, we observe variations of *V*_AB_ as a function of *ν* implying variations of the compressibility of the bulk, even within the quantized Hall plateaus. Below we will discuss the following in more detail: the mechanism leading the observation of *V*_AB_=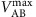 only for a specific subintervals of the Hall plateau and the observed asymmetry with respect to the centre of the plateau.

### Model and further measurements

We base our model on the formation of the compressible and the incompressible strips resulting from electron–electron interactions and the confinement. Since the non-interacting single-particle picture yields a stepwise density distribution violating the electrostatic equilibrium^10^, it is propounded that the 2DES comprises local insulator-like incompressible regions surrounded by metal-like compressible regions. Strips with integer *ν* emanate from direct Coulomb (Hartree) interaction, whereas, the strips assuming fractional *ν* stem from many-body interactions. Taking into account interactions and the lateral confinement of a narrow Hall bar results in incompressible region(s) in its bulk, or alternatively at its edges, alternating as a function of *B*. The spatial distribution and the widths of the incompressible strips are determined by the external *B*, the density gradient, the temperature and most importantly by the energy gap^10^,^11^. Assuming a constant *E*_F_, the evolution of the incompressible regions with changing *B* field can be summarised as follows: In the limit of a high field with *ν*<2 only the lowest Landau level is partially filled (assuming spin degeneracy). Due to the lateral confinement, the spatial distribution of the electron density is inhomogeneous, such that it reaches its highest value close to the centre (at the bulk) whereas it gradually decreases towards the edges and vanishes at the boundaries. Therefore, also *ν* is largest in the centre and decreases towards the edges.

**Figure 2:**
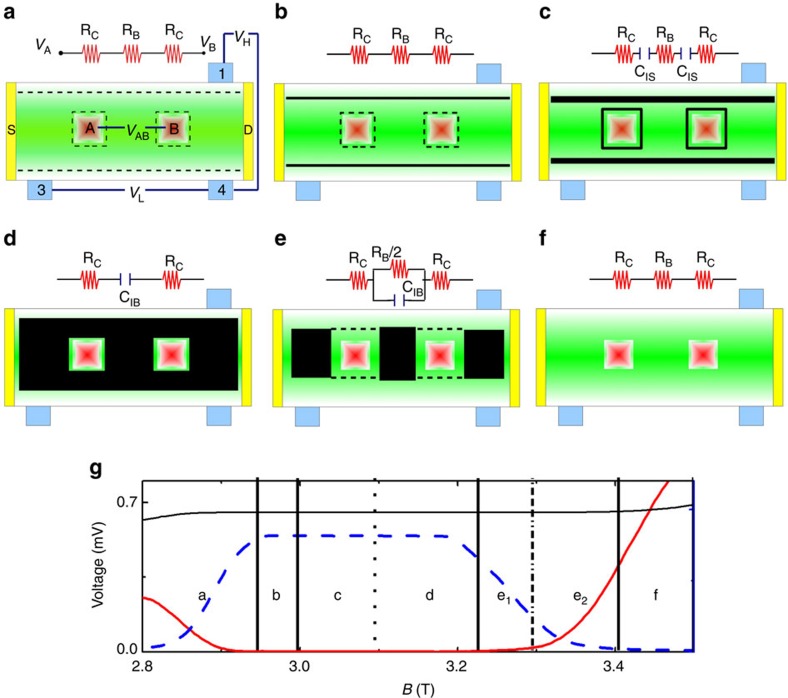
Graphical sketches of the electron density together with the measured voltages. In graphical sketches (**a**–**f**) green colour gradient depicts the electron density distribution at compressible regions, whereas black regions show incompressible regions and dashed lines correspond to thermodynamically compressible strips. Boxes denote different contacts such that colours identify, blue: probe contacts, red: inner contacts and yellow: source and drain contacts. At the top of each sketch the corresponding electric circuit of each situation is shown, where *R*_*C*_ and *R*_*B*_ denote the contact and compressible bulk resistances respectively, while *C*_*IB*_ depicts the bulk capacitance and *C*_*IS*_ the capacitance of the encircling strips. The measured voltages *V*_H_ (solid thin black line), *V*_L_ (solid thick red line) and *V*_AB_ (broken blue line) are shown in **g**. Here the vertical lines are guide to eye to separate different mechanisms depicted in **a**–**f**.

At a somewhat smaller *B*, the *E*_F_ falls into the Landau gap in the centre, yielding an incompressible bulk with *ν*=2. This resembles the single-particle description of the IQHE, where *V*_H_ is quantized and *V*_L_ vanishes. Further decreasing *B* results in a situation where the incompressible region with *ν*=2 shifts towards the edges where it shapes as narrow strips. The evolution of the incompressible strips as a function of *B* is well understood both experimentally and theoretically^11^,^13^, and results in the quantized *V*_H_. Physically, scattering between opposite edges is prohibited by the incompressible regions between them. Here we used the fundamental principle of thermodynamics in determining the (in)compressibility of the strips: In the thermodynamical approach, quantities are physically meaningful if there is a sufficiently large number of particles, so that one can still use the Fermi distribution function. At *T*→0, where only electrons at *E*_F_ contribute to current, this thermodynamic consideration is meaningless at length scales below the Fermi wavelength *λ*_F_. From this thermodynamical point of view it is reasonable to expect that incompressible strips narrower than *λ*_F_ becomes transparent due to scattering between neighbouring compressible regions^18^. In this situation, *V*_L_ becomes finite and *V*_H_ deviates from its quantized value. The schematic presentation of the above discussion is depicted in Fig. 2a–f, starting from low magnetic field strength. We depict the local filling factor distribution of a Hall bar with two inner contacts, where *B* increases through (a) and (f). The electron density gradient is depicted by the green colour gradient, whereas the incompressible regions are shown in black. Similar to edges, incompressible strips form in the close vicinity of inner contacts, where broken lines denote the situation where the strips become leaky (semi-transparent) both thermodynamically and electrically.

In the following discussion we will assume spin degeneracy initially, since the mechanism to elucidate experiments is independent of the particular nature of the energy gap. We start our investigation with the clearest situation where only the lowest Landau level is partially occupied, that is, the 2DES is completely compressible as depicted in Fig. 2f, and proceed our discussion lowering the magnetic field till the system essentially becomes compressible again as shown in Fig. 2a. In Fig. 2g we present the measured potential difference between the inner contacts *V*_AB_ (broken blue line), the Hall potential *V*_H_ (solid thin line) and the potential difference between contacts 3 and 4 (thick red solid line), *V*_L_. In the graphical demonstrations (a–f) the electron density variation is depicted by the colour gradient (green), which vanishes (white) near both the inner (red square boxes) and the source-drain contacts (yellow, rectangular boxes). The black coloured areas correspond to incompressible (constant density) regions. It is worth to note that, sketching the density gradient and its behaviour near the contacts is well justified both experimentally and theoretically^17^,^19^. We see that for *B*≳3.4 (panel label f) *V*_H_ and *V*_L_ increase (almost) linearly, whereas, *V*_AB_ is considerably small. This behaviour suggests that the bulk of the 2DES acts as a poorly conducting metal, as predicted. We explain the behaviour of *V*_AB_ by modelling the bulk as serially connected resistances, composed of the inner contact resistance(s) *R*_*C*_ and the resistance of the 2DES between contacts *R*_*B*_. Decreasing the magnetic field, results in the quantization of *V*_H_ and an increase in *V*_AB_, whereas *V*_L_ remains non-zero (e). We elucidate these behaviours as follows: the region between contacts 1 and 4 becomes incompressible and decouples the Hall contacts and yielding a perfect quantization. However, due to the density gradient induced by the inner contacts, the entire bulk is not incompressible. We model this situation by a resistance and a capacitor (*C*_*IB*_) connected in parallel. Since the capacitance approaches zero for an ideally clean sample at *T*=0, due to *D*_T_ (*E*_F_)=0, all the measured potential results from *R*_*B*_ and *R*_*C*_. In addition there are regions between the contacts 3 and 4 where scattering takes place, yielding a non-zero *V*_L_. Notice that, *V*_L_ approaches zero in the e_1_ region since the resistance along the leaky incompressible strip is reduced compared to a fully compressible bulk, as in the e_2_ region. Lowering *B* further, results in formation of a bulk incompressible region, an insulating state, spread all over the sample: region d. Here, QHE is well developed and *V*_AB_ becomes remarkably large and is bound by a cutoff voltage, which we attribute to finite TDOS at *E*_F_. Namely, since *V*_AB_=*IZ*, where *I* is the excess current, the impedance reads 
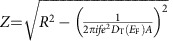
=constant. At lower *B* the bulk incompressible region splits into strips, two of which reside along the edges and the other two encircling the inner contacts. The edge strips decouple Hall contacts and, simultaneously, the encircling strips decouple the inner contacts due to their approximately infinite capacitance, resulting in a constant *V*_AB_, known as the Corbino effect^20^. Note that, from the plotted measurements one cannot quantitatively determine the boundary between (c) and (d), however, when we discuss the temperature effects this transition will become clear. As a remark, note that the centre of the Hall plateaus and 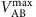 do not coincide. The cardinal situation is where the encircling strips become thermodynamically compressible, that is, similar to a leaky capacitor depicted by broken lines in panel e_1_, whereas the edge strips are still well decoupling as shown in Fig. 2b by solid strips. In short, the evanescent encircling strips can be considered as leaky capacitors and the impedance between inner contacts is modelled similar to that of situation (f). Compared with the inner contacts, the incompressible strips at the edges are sufficiently wide, due to a smoother density profile there decoupling Hall contacts, yielding a plateau. As the edge strips connect contacts 3–4 *V*_L_ is zero. In region (a) the edge strips also become transparent marking the transition between the Hall plateaus. Now, we can answer the questions asked at the end of the previous Section. *V*_AB_ is constant only in a certain *B* subinterval since the inner contacts are only decoupled by the incompressible bulk (panel d) or by the encircling incompressible strips (panel c) for a specific *B* interval and the observed asymmetry is a direct result of incompressible state distribution depending on *B*. Namely at its low field side the constant *V*_AB_ is caused by encircling strips whereas at its high field side it emanates from the incompressible bulk region. Our model also predicts the same behaviour for *ν*=1 and 2/3, if by the assumption of spin degeneracy results in a Zeeman gap for *ν*=1 and assume a many-body gap for *ν*=2/3. Interestingly, for all other visible plateaus we do not observe the saturated *V*_AB_, which suggests that the 2DES does not comprise an entirely incompressible bulk. Note that, for *ν*=3 and 4 the maximum value of the spikes just hits 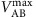.

### Measurements at varying temperature

**Figure 3:**
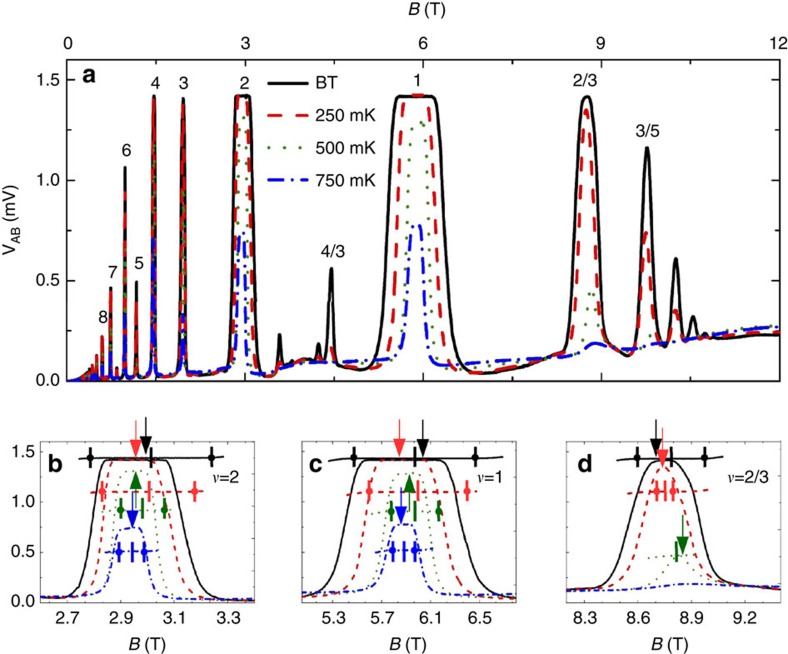
The temperature dependency of Hall potential and the inner voltage difference. The 12 Tesla wide span of *V*_AB_ for various temperatures is shown in **a**, where the Source-Drain excitation is *V*_SD_=26 mV and the excitation applied between inner contact A and B is *V*_IN_=4 mV. Here we used sufficiently small excitation voltages (corresponding to sample current below a nano ampere) to prevent additional Joule heating stem from scattering processes. In panels **b**–**d,** we focus on characteristic magnetic field intervals such that the electronic system is in a quantum Hall state, that is, *ν*=2, 1 and 2/3 respectively. The quantum Hall plateau intervals are depicted by vertical thick lines with filled-dost at their centres for each temperature, namely black (base temperature), red (250 mK), green (500 mK) and blue (750 mK). The thin vertical lines show the centre of Hall plateaus, whereas arrows point the centre of constant potential interval observed at the inner contacts. Both are obtained by the half-length of the relevant magnetic field interval.

As the incompressibility conditions of the edge or encircling strips are critical to determine the transition from situation (c) to (d), it is worth to investigate the effect of temperature on the observed features, by which we can also test our constant TDOS argument. One expects that, an increase in *T* will result in a decrease of 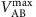 which indirectly measures the available states at *E*_F_. Since an increase in temperature will elevate the number of available states at the *E*_F_ and the neighbouring Landau levels will overlap stronger as direct consequence, the quantum capacitance will increase and the impedance will decrease. In addition, due to thermal broadening of the Landau levels the incompressible regions will shrink in their widths at higher temperatures, hence the regions in *B* of 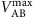 will also shrink. This will allow us to determine qualitatively the transition between situations (c) and (d). Figure 3 plots the temperature dependence of *V*_AB_ together with *V*_H_. The experimental findings strongly follow our expectations, such that the saturation value decreases (∝1/*D*_T_ (*E*_F_)) meanwhile subinterval shrinks till the thermal energy overcomes the energy gap. We can identify the largest incompressible bulk at *B*∼2.9 T for *ν*=2 from 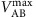, where for elevated temperatures the centre of *V*_AB_ and *V*_H_ now coincides and pins the largest bulk incompressible region. The relative centres (and widths) of the Hall plateau (perpendicular lines) and *V*_AB_ (arrows) are shown in Fig. 3b. One can see that the centre of the Hall plateau shifts to lower *B* values and coincides with the centre of *V*_AB_. Note that, the encircling strips decay faster compared with edge incompressible strips, since the density gradient is larger in front of the contacts with respect to edges^17^. Therefore, due to thermal broadening of the TDOS, electrons can scatter across the incompressible strips, hence become leaky. A similar line of argumentation also holds for *ν*=1, however, for the focused fractional state *ν*=2/3 the maximum shifts to the high field edge of the plateau interval. We attribute this effect to differences in the activation behaviour of the many-body gap. The temperature dependency of *V*_AB_ completes our experimental investigation.

As a final remark, although our samples are of high purity, one should keep in mind that disorder always leads to localized states at the tail of the Landau band. In fact, this is reflected in our results by the finite (and constant) plateau value of the 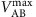, which essentially gives the thermodynamical DOS at *E*_F_ within the quantized regime. If one repeats similar experiments using samples with a higher degree of disorder, the localised states at *E*_F_ will dominate the transport. Then, the quantization interval and the constant *V*_AB_ interval will overlap fully, since all the bulk will become localized, that is, insulator, and the localization picture of the QHE will be recovered.

We have reported on the bulk transport measurements of a topologically modified Hall bar. We have experimentally evidenced that it is possible to observe the quantized Hall effect even if the bulk is not entirely incompressible. Quantized conductance is then guaranteed by incompressible strips residing at the edges and preventing scattering between the Hall contacts. We also find that the incompressible bulk based topological theories are well justified at certain magnetic intervals for finite size and non-periodically bound systems. In addition we showed that, the incompressible strips narrower than the thermodynamical length scales are prone to become leaky in the plateau-to-plateau transition intervals. Note that the Berry flux enclosed in real-space comprised by the incompressible strip equals to the number of fully occupied Landau levels in momentum space, hence, the Chern number is still determining the Hall conductance. Moreover, the temperature-dependent measurements clearly indicate that the capacitive coupling of the inner contacts goes beyond the trivial cross-capacitance at low temperatures. The experimental results outlined here and analysed based on the screening theory shows explicitly that the bulk of the sample in real-space is compressible for specific field strengths. Hence, it is important to understand the momentum-space bulk properties to determine interrelation between the topological insulators and quantized Hall effect.

### Data availability

The data that support the findings of this study are available from the corresponding author upon request.

## Additional information

**How to cite this article:** Kendirlik, E. M. *et al*. The local nature of incompressibility of quantum Hall effect. *Nat. Commun.* **8,** 14082 doi: 10.1038/ncomms14082 (2017).

**Publisher's note**: Springer Nature remains neutral with regard to jurisdictional claims in published maps and institutional affiliations.

## Supplementary Material

Supplementary Information Supplementary Notes, Supplementary Figures and Supplementary References.
