# Supplementary material for: The local nature of incompressibility of quantum Hall effect
Source: Nat Commun. 2017 Jan 10;8:14082. doi: 10.1038/ncomms14082 (PMC5234089; doi:10.1038/ncomms14082)
Supplement: Supplementary Information — Supplementary Notes, Supplementary Figures and Supplementary References. [file ncomms14082-s1.pdf]

Supplementary Note 1. Introduction

In this supplementary material section we will first provide the details of our experimental setup and then discuss the validity of some crucial assumptions of the well known theories of the IQHE. Namely, first we will briefly discuss the assumptions on different boundary conditions and topological aspects of the quantised Hall samples within the single-particle (SP) theories. The main discussion is on the mapping between the momentum space representation of the edge states to real space representation. This discussion clarifies the importance of boundary conditions, where the normalisation condition is the only physical restriction. Here, instead of a detailed mathematical description we will make use of some schematic presentations which are common in the QHE discussions. However, to lift the confusion between different theories we used distinguishing colours to discriminate single-particle and screening theory concepts, e.g. incompressibility, edge states, etc.

Our first discussion is based on the single-particle theory, where incompressible (namely energy gapped region or so to say insulating) bulk is essential for QHE and the incompressible regions are denoted by blue colour. We denote the SP chiral edge states by solid lines, which differ by colours depicting different filling factors, e.g. red corresponds to $\nu = 2$ edge state where spin degree of freedom is neglected. On the other hand, consistent with the main text, we denote compressible 2DES by green and incompressible 2DES by black, within the screening theory. Yellow regions denote the contacts, whereas white corresponds to electron depleted (etched) regions for both theories.

First, we aim to clarify that, using periodic boundary conditions for finite size Hall bars is questionable which then makes topological arguments prone to break. Second we would like to highlight that, considering quantum capacitance in calculating impedance or current modifies both the SP and screening theory results. And third, we explicitly show that our sample geometry is quite different then the well known Corbino and the anti-Hall bar geometries.

Supplementary Note 2. Experimental Setup

We show the 3D schematic representation of our sample in Supplementary Figure 1 and the equivalent circuit diagram of our inner contact measurements in Supplementary Figure 2. We excite the inner contacts using the internal oscillator of the SR 850 lock-in-amplifier imposing a 4 mV ac voltage in series with a 10 MΩ resistor, hence driving a constant current as explained below. First we have to clarify that if the contact impedance of A (or B) approaches or exceeds the input impedance of the preamplifier (LNP) (100 MΩ), then our results might become unreliable. However, note that the current flowing from inner contacts is approximately 0.4 nA, hence, from the measured V_{AB} one can assume that the maximum impedance of the sample is nearly 3.5 MΩ, well below the input impedance of the preamplifier. Hence, we expect that the equivalent circuit will be a voltage divider between the sample and other circuit elements. This is essentially very close to what we observe: once 4 mV is imposed, the maxima of V_{AB} is similar to 1.4 mV, which indicates that the rest of the voltage is dropped at the other circuit elements. That is, say for 4 mV excitation 2 mV will drop at the sample and 2 mV will drop at the other circuit elements. The coaxial cable capacitances are of the order of few hundred pF, whereas cable resistances are of the order of few hundred Ohms. The capacitance between the inner Ohmic contact and the 2DEG for sure also contains both the classical (geometrical) and the quantum mechanical part. The depleted electron region in front of the contact of course is an insulator and is added to our model. Note that, the contact capacitance is almost always constant while sweeping magnetic field and is also sufficiently small. A simple model of the contact capacitance can be described by $C_{\mathrm{oc}} = \epsilon L/2\pi^2 \ln(4d/l_d)$, here L is the perimeter of the Ohmic contact, d is the distance between the contact and the 2DES and l_d is the electron depleted region in front of the contact. Assuming that the 2DES is 100 nm below the surface and the depleted region is at the order of 100 nm, the contact capacitance just depends on the perimeter, which is approximately 4 μm. The resulting capacitance is at the order of 10^{-11} F.

Estimation of quantum capacitances

As mentioned in the main text, the quantum part of the capacitance is directly related with the thermodynamic density of states (TDOS) of the system. The TDOS is obtained as,

$$D_{\mathrm{T}}(\mu, T) = dn_{\mathrm{el}}/d\mu, \tag{1}$$

where, T is temperature, μ is the chemical potential and n_{el} is the average electron density. One can obtain the electron density within a mean field approximation as,

$$n_{\mathrm{el}}(\mu, T) = \int_{-\infty}^{\infty} dE f(E; \mu, T) D(E), \tag{2}$$

using the Fermi function $f(E;\mu,T)$ and density of states $D(E)$. Assuming bare Landau density of states, $D(E) = \frac{1}{2\pi l^2}\sum_{n,\sigma}\delta(E-E_{n,\sigma})$, where n is the Landau index and σ indicates spin degree of freedom, one obtains the TDOS of a 2DES subject to magnetic field as,

$$D_{\mathrm{T}}(\mu,T) = \frac{1}{\pi l^2}\sum_n \frac{\beta}{4\cosh^2(\beta[E_n-\mu]/2}, \tag{3}$$

β being the inverse thermal energy, i.e. $1/kT$. As a first order approximation and considering the lowest Landau level one obtains,

$$D_{\mathrm{T}}(\mu,T) = \frac{1}{\pi l^2}\frac{1}{kT}. \tag{4}$$

Assuming the B field to be at the order of 10 T and temperature to be 100 mK, one calculates the quantum capacitance $C_q = e^2 D_T.A$ for an area of 1 μm^2 to be at the order of few nF, which depends on the temperature and the precise shape of the Landau level broadening. A very important experimental finding is the "Background DOS" between Landau levels, observed by D. Weiss and co-workers , where the localised states at the tails of Landau levels are observed clearly [1]. Note that, a back of envelope calculation yields 10^{-18} F for the quantum capacitance if one assumes vanishing temperature and no level broadening, hence one can safely ignore the classical capacitance of the Ohmic contacts. Here we assumed the constant DOS ($D_0 = 2.8\times10^{10}$ meV^{-1} cm^{-2}) and the area of the contact to be 1 μm, in principle with magnetic field DOS will be at the order of 1 percent of D_0. Therefore the contribution of the lead capacitance to total impedance can be thought as a small background signal. The cable capacitances can be obtained by the rule of thumb that (for coaxial cables) 1 pF per cm, since our all lines were coaxial cables we have cable capacitances at the order of few tens of pFs. Again negligible compared to the quantum capacitances.

We should also clarify that there are two grounds and how one can be sure that which current goes to which contact. In fact, while performing measurements we were also concerned with the same question, hence, performed experiments either without imposing voltage excitation to SD contacts or to the inner contacts AB, which is mentioned in caption of Fig1. Also note that, while measuring only with inner contacts S and D contacts were left floating and vice versa. As one can see from Supplementary Figure 3, if the D contact is also grounded almost no cross current flows from the SD to AB contacts. For sure, at higher current densities this would be drastically changing, due to sample heating. Over all, we checked the possible complexity of current distribution and can ensure that the experimental results are sufficiently safe to draw conclusions. At this point we would also like to remind that, the SD and AB contacts are excited with different frequencies, hence, we expect to measure only the voltage drop at the bias frequency. Namely, the voltage drop at the inner contacts is at 11.4 Hz and the SD voltage is at 8.54 Hz. For sure there is a possibility of cross-scattering which is rather small as it can be seen from Supplementary Figure 3. However, at higher currents (>10 nA) this cross-scattering yields exciting

breakdown effects, which are to be discussed in an upcoming publication. In the same figure we also show the relative phase shifts of the resistive and capacitive parts measured by the LIA, which clearly assures the capacitive coupling of the 2DES to the measurement device.

Supplementary Note 3. Physical boundary conditions and their relation with Topology

Once the Landau Hamiltonian is solved using Coulomb (also called Landau) gauge, i.e. translational invariance in y direction and open boundary conditions in x direction ($\Phi(x \to \pm\infty, y) = 0$), the solution yields plane waves in the current (y) direction as if as a free electron and harmonic oscillator wave functions in the other direction. Such a choice of gauge and boundary conditions can be utilised to describe a homogeneous Hall bar that extends to infinity in both directions. However, to describe a more realistic Hall bar one usually assumes infinite walls in x direction, which modifies the related wave-functions by parabolic cylinder functions approaching to simple harmonic oscillator solutions away from the boundaries. However, these preferences of boundary conditions yield the problem of normalisation. In order to overcome the normalisation problem one assumes periodicity in momentum along the current direction similar to Bloch wave function describing electrons in a crystal. As the Hall bar is not periodic in real space, one cannot impose periodicity in y, hence, periodicity in k_y is assumed. This assumption yields to the well known description of Thouless [2] which explains quantised Hall effect in terms of Chern numbers in *momentum* space, where one simply counts the Berry flux encircled and describes transport utilising the Kubo formalism. Such an approach is well justified only if the system is in electrostatic equilibrium, i.e. if no external current is imposed, or might be reasonable if the QHE can be treated within the linear response regime, if it can be handled within this regime at all.

The first case can be ruled out in our experiments since we excite the system by an external voltage. The second case is also ruled out, since the excitation energy (voltage) is comparable with the Landau gap, hence the system is far away from linear response. In contrary to periodicity in momentum space, Laughlin assumed periodicity in real space considering a cylinder where instead of an imposed current he assumed a radial magnetic field which changes adiabatically by time (cf. Supplementary Figure 3), leading to a phase which is protected by the topology resulting in conductance quantisation. [3] This approach also for sure does not entirely describe the experimental realisation of the QHE, at least for our experiments. Both of the descriptions of the QHE implicitly require a bulk incompressibility, namely an energy gap induced by the magnetic field. This is somewhat similar to 3+1 D topological insulators where an energy gap opens due to crystal structure and provides an incompressible (insulating) bulk. [4] However, note that this gap is in the energy dispersion, namely when one plots the relation between the energy and momentum there opens a gap due to symmetries of the crystal. Similarly, for an infinite Hall bar with periodic boundary conditions one can obtain such a gapped energy dispersion in momentum space, which is usually mapped to real space without taking care of the boundary conditions of the physical devices. For a finite width and length (flat) Hall bar assuming periodic boundary conditions is not appropriate. Hence, bulk incompressibility which is essential to describe QHE does not

apply to our physical system at hand. In connection with 3+1 D topological insulators, we should note that in this case the system is periodic in (crystal) momentum space and hence the energy dispersion presents the well appreciated "topologically" protected energy gap. In contrast, QHE does not provide the periodicity in momentum space once realistic, i.e. physical, boundary conditions are considered.

Supplementary Note 4. Real space-momentum space duality of Halperin-Büttiker edge states

The first important manifestation of boundary conditions on the transport properties of a 2DES subject to high magnetic fields is proposed by Halperin in early 80's. [5] In his pioneering work he imposed the above mentioned infinite wall boundary conditions in the radial direction r, which essentially bends the Landau levels in the close vicinity of boundaries. Utilising periodicity in azimuthal (θ) direction (both in momentum and real space), one can map the energy dispersion (i.e. energy versus momentum) to real space (i.e. energy versus r) and obtain the geometry known as the Corbino geometry, as shown in Supplementary Figure 5. In the original work it is stated that this is a "slight modification" of Laughlin's cylindrical geometry (see Supplementary Figure 4), which is not reasonable from topological point of view in real space, since the genus numbers are different (for Laughlin's case it is 1, since periodicity is also assumed in z which defines essentially a torus, and for Halperin's case it is 0). We should also note that, due to the absence of edges in Laughlin's geometry, the current is uni-directional, whereas at a Corbino edge states are present and carry chiral current. For a Corbino geometry, in contrast to a Hall bar (cf. Supplementary Figure 5), there are no source and drain contacts, however, the edge states exist due to boundaries where all the system is in equilibrium. Namely, no external current can be and is imposed. In this geometry, one can measure the conductance between the inner and outer contacts if contacts are kept at different electrochemical potentials. In such a system the electrical measurements essentially results in a quantised Hall conductance. It is also stated that "In a real experiment, the measured Hall potential eV is the sum of an electrostatic potential eV_0 and the difference in Fermi levels $E_F^{(2)} - E_F^{(1)}$ (electrochemical potential difference in our notation). The edge current is then only a *fraction* of the total Hall current, given by

$$(E_F^{(2)} - E_F^{(1)})/eV \approx \alpha n r_c \hbar\omega_c C/e^2, \tag{5}$$

where C is the capacitance per unit length of the edge states, and α is a number of unity.". Here, n is an integer determined by the filling factor ν at the bulk and Fermi energy lies in between the energies E_ν of two Landau levels $\nu = n-1$ and $\nu = n$, in the interior of the sample. The electrochemical potentials near the boundaries ($E_F^{(2)}$ and $E_F^{(1)}$, at r_2 and r_1, respectively, where the contacts reside) are also supposed to lie in the interval $E_{n-1} - E_n$, which implicitly assumes linear response. Then the total current carried by the edge states between $E_F^{(2)}$ and $E_F^{(1)}$ is given by $neh^{-1}(E_F^{(2)} - E_F^{(1)})$. Now, we should clarify couple of points with the properties of contacts and their equilibration with edge states: first, in Halperin's approach it is implicitly assumed that the capacitances C_1 and C_3 shown in Supplementary Figure 5a are neglected, which imposes that the contacts are in electrochemical equilibrium with the edge states, namely the scattering between the edge states and contacts is possible pointing a compressible edge. Hence, the contribution to Hall current in real experiments only come

from the bulk capacitance, which is quantised if finite TDOS at E_F is assumed. If the compressible edge assumption is lifted, then the total capacitance would be the sum of C_1, C_2 and C_3. For $\nu_{centre} = 2$ the total capacitance would yield an edge current approximately $\frac{2}{3}\alpha r_c \hbar\omega_c C/e^2$, which is not quantised. Here we assumed $D_T(E_F^{(1)}) = D_T(E_F^{(2)}) = D_T(E_F)$. For higher bulk filling factors, such an approach implicitly assumes that the inner edge states are in equilibrium with each other similar to the outer edge states, which again requires that the edges are compressible, i.e. scattering between inner or outer edge states is possible. This assumption then makes the current quantisation questionable.

As discussed in the main text and details given in the following Subsection, capacitance is composed of the classical and the quantum counterparts. Once, the region between two edge states (or contacts) is insulating (namely incompressible) then the quantum capacitance vanishes for an ideally pure system. Hence the Hall current mentioned above reads to zero, i.e. impedance diverges. In real experimental devices there are potential fluctuations due to disorder which yield localised states as discussed by Halperin, which then results in conductance quantisation for a finite magnetic field interval, where bulk incompressibility is still preserved.

Furthermore, once again the main assumption is the incompressibility of the bulk, i.e. the impedance between the inner and the outer contacts reads to infinity (or relatively high compared to conductance) hence the conductance measured between these contacts should be quantised. To be explicit, if one wants to move an electron from one contact to the other one has to get across n edge states (namely pass through metal-topological insulator boundary n times) and has to pay an amount of energy that corresponds to n (Berry) flux quanta, yielding to quantisation. In Supplementary Figure 5 we present both the Hall bar and the Corbino geometries also depicting "edge states". Note that, in the Corbino geometry edge states are parallel to the contacts whereas for the Hall bar geometry they are perpendicular to the contacts. Hence, the impedance is infinite (or huge for real devices at finite T) for the Corbino geometry, however, for the Hall bar capacitance vanishes (or much smaller then the resistance) considering a "Büttiker" contact, i.e. transmission from the contact to edge state is unity.

It is important to note that, the mathematical mapping between the momentum and real space representations of the edge states makes sense only if the periodicity is preserved, which is the case for the Corbino geometry. However, when a finite size Hall bar is considered such a mapping becomes questionable, at least for all geometries.

Couple of years later than the work of Halperin, Büttiker developed a transport theory based on the Landauer formalism to describe the IQHE. [6] In his work also the finite size of the Hall bar is taken into account by imposing a confinement potential in x direction, which essentially varies smoothly on quantum mechanical length scales. Although yielding to similar results with Halperin, the smooth confinement potential allows one to use simple harmonic oscillator solutions also close to the boundaries. In addition, the scattering probability

between edge states at the same boundary is suppressed by the fact that the edge states are relatively far apart from each other both in real space and in momentum space. Utilising the periodicity in momentum space in equilibrium one can then map the momentum space representation of edge states to real space and draw the well known "Büttiker" edge states for a Hall bar as shown in Supplementary Figure 5b. This picture is modified if an external current is imposed by applying a voltage difference ΔV between the source and drain contacts, yielding an electrochemical potential energy difference $\mu = eV$. Of course in this non-equilibrium situation assuming periodic boundary conditions become questionable, where one end of the Hall bar is kept at potential V. Turning back to our discussion on the capacitances and calculating the actual Hall current one can still utilise Eq. 5. Note that the edge states at the same sides have the same electrochemical potential, hence, C_1 and C_3 vanishes and the only contribution to capacitance comes from C_2. Once again, for an ideal 2DES capacitance vanishes yielding zero Hall current pointing a back-scattering free transport throughout the plateau interval. However, even a small amount of TDOS below E_F would yield a finite capacitance, hence a finite Hall current which would result in deviations from perfect quantisation.

By the above discussion, we have shown that the momentum-real space duality of both Halperin and Büttiker edge states becomes questionable since such a duality strongly depends on the imposed boundary conditions and the symmetries of the sample. In addition, once the quantum capacitance is taken into account, which is directly proportional to TDOS at E_F, impedance between probe contacts also strongly depends on the edge state configurations, namely whether the edge states are perpendicular or parallel to imposed current. Such geometrical and topological aspects of so called QHE samples are usually undermentioned in the well known theories.

Supplementary Note 5. Classical and Quantum capacitances between contacts

In a simplistic treatment, inverse capacitance can be described as the amount of energy to add a charge to a system, given by $Q^2/C_c = E$. Classically, one can approximate the capacitance of a sheet as shown in Supplementary Figure 6 as $C_c = \frac{\epsilon L}{2\pi^2} \ln \frac{4d}{w}$, where ϵ is the dielectric constant, L is the perimeter of the ring, w is the width of the incompressible strip and d is the distance between the gate and the 2DES. [7] On the other hand, the quantum capacitance per area is solely dependent on the thermodynamical density of states given by $C_q = e^2 D_T(E_F)$. Since these two capacitances are connected in series, the total capacitance is given by $1/C = 1/C_c + 1/C_q$. The classical capacitance is finite except that the width of the incompressible region is infinite, however, the quantum capacitance becomes zero if there exists an incompressible strip between the inner and outer contacts at zero temperature and for an ideally clean system, i.e. no level broadening due to impurities. Therefore, the capacitance is dominated by the quantum counterpart if there exists an incompressible strip decoupling the contacts. Note that, the contacts and the compressible regions are in electrochemical equilibrium, since they both behave like a metal. Furthermore, since scattering is suppressed exponentially along the incompressible ring and is quantised across the ring, resistance is much smaller than the total capacitance therefore impedance reads

$$Z \propto 1/D_T(E_F) \to \infty. \tag{6}$$

Hence, for a Corbino geometry the bulk incompressibility is the guarantee of conductance quantisation within the single particle picture. However, when interactions are taken into account we observe that it is not necessary to have an incompressible bulk and only a single incompressible strip is sufficient to decouple inner and outer contacts.

The next discussion is on the thermodynamical definition of incompressibility which is a statistical quantity. One usually defines a system to incompressible if the ratio between the change in number of particles and the change in electrochemical potential presents a discontinuity. Note that, electrochemical potential is a statistical quantity which makes sense physically only if there are sufficient number of particles within the system considered. Therefore, assigning incompressibility to a strip is bounded by the number of particles within the strip, which is essentially determined by the Fermi wavelength at zero temperature. Hence, once the strip width becomes small or comparable with the Fermi wave length the insulating behaviour of the strip is destroyed. Such a situation can be considered as a leaky capacitor, as stated in the main text. In addition, when the strip width becomes even smaller than the magnetic length, which is the quantum mechanical length scale, then it is possible to tunnel across the incompressible strip by quasi-inelastic scattering mechanisms.

Supplementary Note 6. What to expect within the Büttiker edge state picture between inner contacts

As discussed above, once the QHE is well developed namely scattering is suppressed by the incompressible bulk the impedance between contacts should diverge for an ideally pure 2DES. Hence, if Hall voltage presents quantisation V_{AB} should diverge simultaneously. However, this is true only at zero temperature and for an ideally pure 2DES. Once, effects of finite temperature and TDOS at the Fermi energy is considered then the Hall plateau shrinks together with the high impedance interval. Let us first consider $T = 0$ however assume a non-ideal 2DES, i.e. with impurities leading to level broadening, then the amplitude of the impedance (therefore V_{AB}) is bounded from up by the TDOS at E_F. Supplementary Figure 7 depicts the upper boundary of V_{AB} at zero temperature by the thick horizontal dotted line, whereas ideally pure 2DES case is shown by the solid (blue) line. Once the impurity scattering is taken into account at finite temperature the curves are modified, which are depicted by the thin broken lines for V_{H} and by the thin solid lines for V_{AB}. Based on above arguments one can conclude that our experimental findings do not coincide with the results expected from Büttiker type edge state theory, up to our knowledge. We show the expected edge state distribution and corresponding capacitances in Supplementary Figure 8. The scattering between edge states out of the plateau interval is depicted by R denoting the resistance. Here one can see once more the geometrical difference between the Corbino geometry (one hole) and our device (two holes). In the latter both contacts are encircled by the edge states where excess current is perpendicular to the contacts, whereas in the former one the outer edge state is encircled by the outer contact and there is no current imposed between the two contacts. The quality of our sample guarantees that the electrochemical equilibration between the edge states is strongly suppressed given the fact that the distance between inner contacts is 3.5 μm, the area of contacts being 1 $\mu\mathrm{m}^2$ and the physical dimensions of the our device is $(W \times L)$ 10×50 $\mu\mathrm{m}^2$. In our sample the mean free path, the localisation and the equilibration lengths are sufficiently large to suppress scattering between edge states both encircling the contacts and the ones along the edges of the sample.

Supplementary Note 7. Differences between Anti-Hall bar and our geometry

Modifying the topology of a Hall bar has been realised some 18 years ago by R. Mani [8] and recently modelled by Oswald and his co-workers within the non-equilibrium network model. [9] At a first glance our experimental system and the so called anti-Hall bar (AHB) geometry seems to resemble each other, as shown in Supplementary Figure 9. However, there are couple of major differences, first at an AHB geometry there is a single etched region at the bulk of the sample. In this configuration edge states percolate around the AHB and contacts are connected by the edge states. However, in our situation we have two electron depleted regions, i.e. inner contacts, which are not connected by incompressible strips. Hence for the AHB geometry, impedance is mainly dominated by the resistive counterpart, whereas for our geometry the transport between contacts is dominated by the capacitive counterpart throughout the plateau interval. Another important difference is the properties of our sample, namely its size and the mobility. Different from the actual AHB sample, our samples are defined on a high purity wafers which strongly suppress scattering between edge states, either between the inner ones or between the inner and outer ones.

Supplementary Note 8. Finite density of states below the Fermi energy, indirect proof of a single incompressible strip and the 1.35 mV value

From the above discussions one can clearly see that the potential difference measured between contacts A and B, should diverge or at least become huge at certain B intervals within the quantised Hall plateau. Consequently, the maximum value of V_{AB} in certain magnetic field intervals is attributed to finite TDOS at the Fermi energy. We also observe that, this maximum value is not bulk filling factor dependent and is not quantised. The non-quantisation is clarified by the temperature dependent measurements. At base temperature and at the lowest inner excitation voltage ($V_{\mathrm{in}} = 4$ mV), we observe that Max(V_{AB})$\approx$ 1.35 mV for the plateaus $\nu = 4, 3, 2, 1$ and $2/3$, whereas it seems that for the other quantised Hall states only a spike is measured. Now let us consider a situation where we imposed an excitation at very low frequencies (practically DC) and let us assume that the an infinite resistor (impedance) is placed between the inner contacts at zero temperature, then we would expect to measure 2 mV, i.e. one has basically 2 to 1 voltage divider. Hence, the maximum value indicates that the bulk is not an infinite resistor, however, it highly decouples inner contacts due to finite TDOS at E_F. In the next experimental investigation we measured V_{AB} as a function of temperature and observed that Max(V_{AB}) decreases by increasing T. The decrease of the maximum value is a direct consequence of finite TDOS at E_F and is a measure of the activation gap, indirectly. In Supplementary Figure 10, we show the Arrhenius plot of Max(V_{AB}) and see the commonly observed activated behaviour. We also checked the value of Max(V_{AB}) at higher excitation voltages and see the same ratio between the Max($V_{\mathrm{AB}}/V_{\mathrm{in}}$), indicating that this maximum value is not due to a cutoff voltage of our electronic setup. Both of these experimental results clarify that the bulk resistance (in fact voltage difference at fixed excitation current) is not quantised. One can also claim that there exists only a single incompressible encircling strip with a given (integer or fractional) filling factor, since if there existed more than one strip Max(V_{AB}) would present a stepwise behaviour at each integer filling factor plateau.

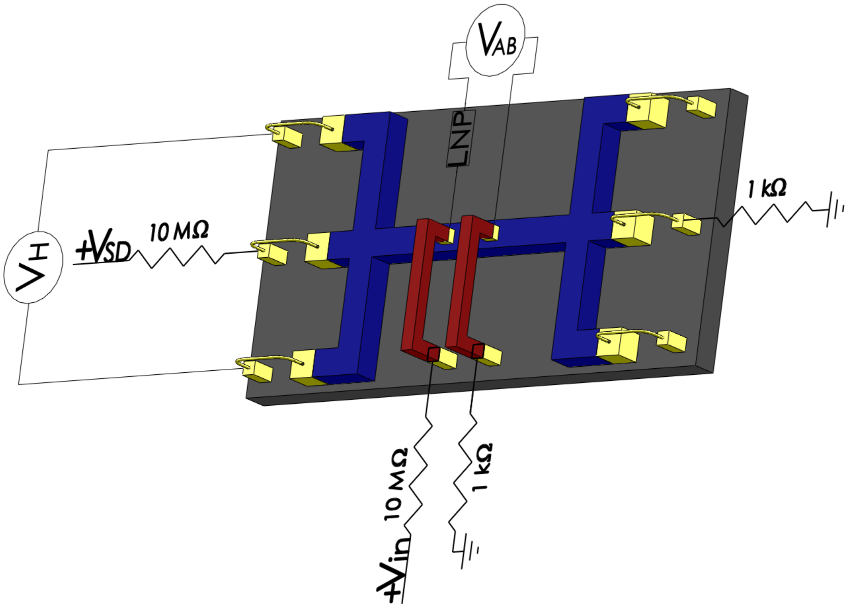

Supplementary Figure 1: Sample sketch. 3D demonstration of our sample geometry, blue areas depict the 2DES, yellow regions are contacts and red denote the air bridges.

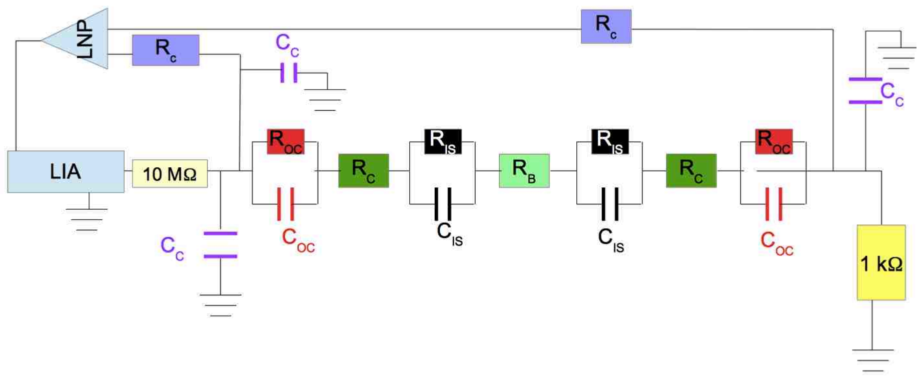

Supplementary Figure 2: Equivalent circuit diagram of inner contact measurement LIA: Lock-in amplifier, input impedance 10 MΩ, output impedance 50 Ω. R_{c}: Cable resistances (of the order of few hundred Ohms), C_{c}: Cable capacitances (of the order of few hundred pF). R_{oc}: Ohmic contact resistance, C_{oc}: Ohmic contact capacitance. R_{C}: Resistance of the compressible region. R_{IS}: Resistance of the incompressible region, C_{IS}: Capacitance of the incompressible region. R_{B}: Resistance of the bulk compressible region. LNP: Low-noise preamplifier (SRS 560), input impedance 100 MΩ, output impedance 50 Ω. LIA: Lock-in amplifier (SRS 850), input impedance 10 MΩ, output impedance 50 Ω.

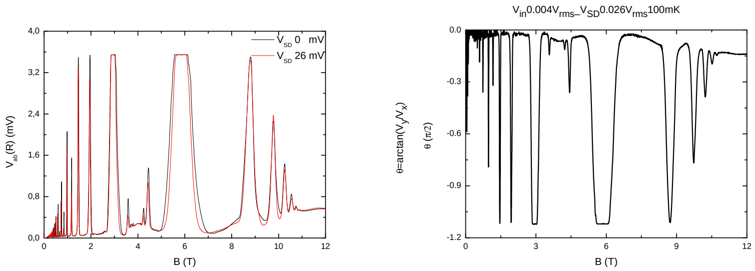

Supplementary Figure 3: Measured voltages and relative phases Panel **a**, plots voltages with and without SD excitation, where one can see no prominent effect of SD voltage on the observed V_{AB}. Panel **b**: The relative phases of the resistive (V_X) and capacitive (V_Y) contribution to measured V_{AB}. The $-\pi$ shift clearly presents the capacitive contribution.

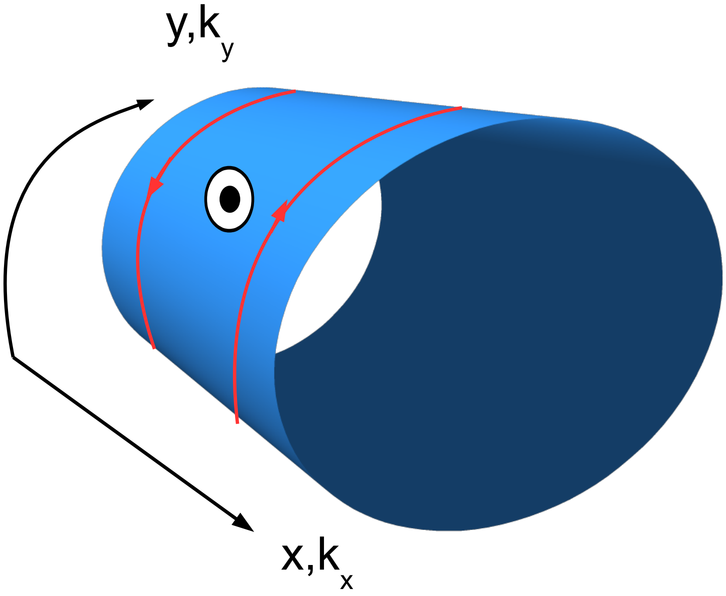

Supplementary Figure 4: Cylindrical geometry. In the earliest attempt to elucidate IQHE, Laughlin proposed that if a 2DES resides on the surface of a cylinder and a time dependent perpendicular magnetic field penetrates the surface, then to protect the geometrical phase only an elementary charge can be moved from one end of the cylinder to the other end when the flux changes by one quantum. In this description periodicity in real space is assumed, however, the boundary conditions are not specified in the perpendicular direction. In contrast, in the approach of Thouless the periodicity is imposed in momentum space k_y, where the 2DES is defined on a flat surface.

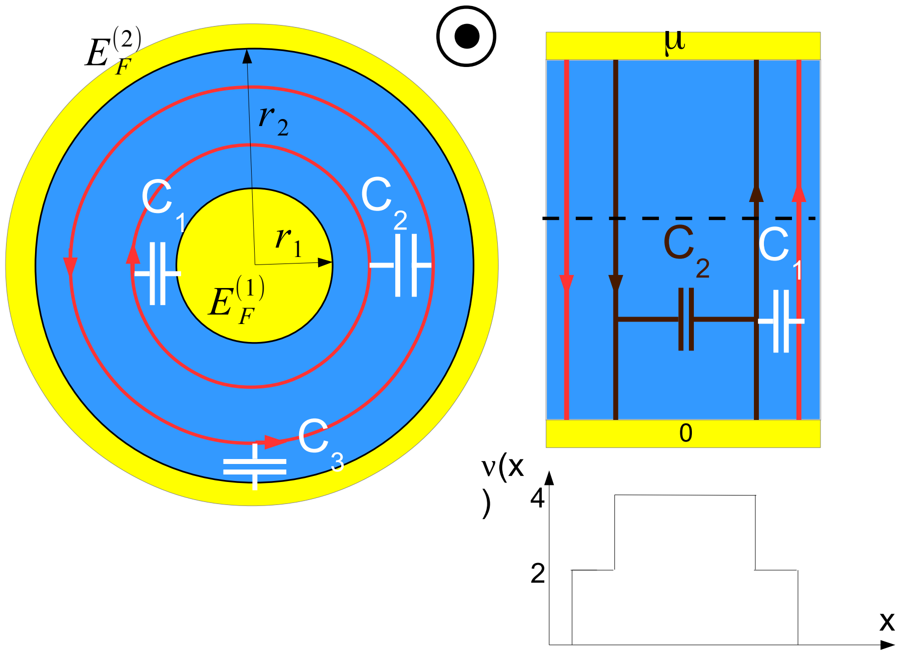

Supplementary Figure 5: The Corbino and Hall bar geometries. The quantised Hall effect measurements are usually performed either on the rotationally periodic Corbino (left) geometry or on the translationally non-invariant Hall bar (right) geometry in real space. For a Corbino disc, inner and outer contacts (yellow regions) are kept at different electrochemical potentials, where edge states (red solid lines) are parallel to contacts and the conductance between these contacts is measured. In contrary, for a Hall bar geometry edge states are perpendicular to the contacts, where contacts are kept at different electrochemical potentials, here 0 and μ. The bottom panel on the right presents the filling factor (or electron density) distribution along the cut denoted by dashed lines in the upper panel. One clearly sees the unrealistic electron distribution which for sure cannot guarantee electrostatic equilibrium. Note that, the electron depleted regions near the edges are neglected, hence the related capacitances, which is consistent with the single-particle theories.

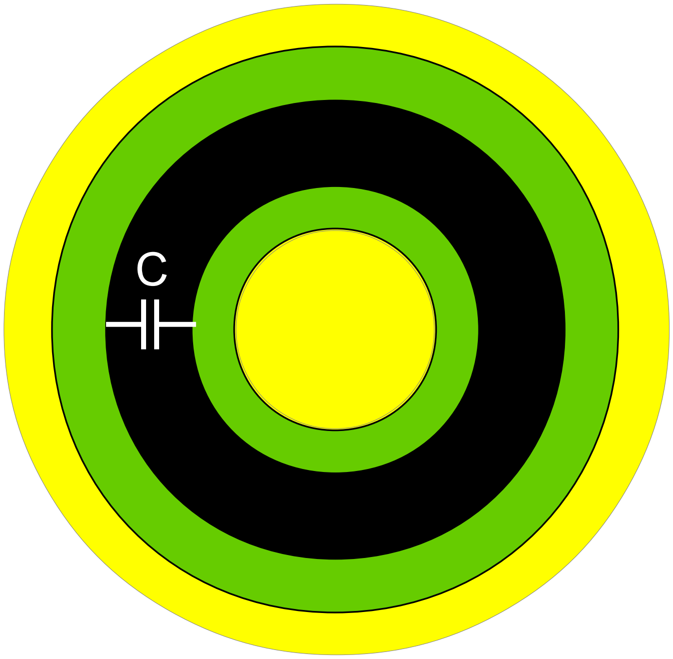

Supplementary Figure 6: Screening theory expectation of a Corbino disc. The inner and outer contacts are decoupled by a bulk incompressible region denoted by black. However, if the magnetic field is reduced the picture essentially does not change drastically. At a lower B field, the bulk incompressible region will split into two encircling incompressible rings near the edges and since the capacitances are connected in series the contacts would be still decoupled.

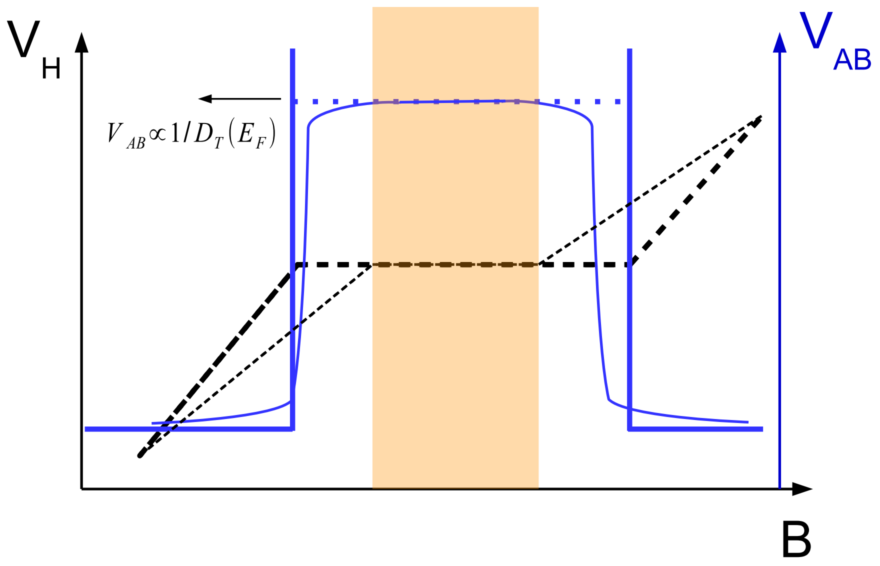

Supplementary Figure 7: The relation between V_{H} and V_{AB} as a function of B field. Based on the above discussion concerning capacitances one expects to observe an infinite V_{AB} whenever the Hall voltage is constant since the bulk should be incompressible. Due to $D_T(E_F) \neq 0$ at realistic samples the impedance becomes finite. In addition, at finite temperatures the sharp features both at V_{H} and V_{AB} are smeared out. However, the constant Hall voltage and high impedance intervals should still overlap.

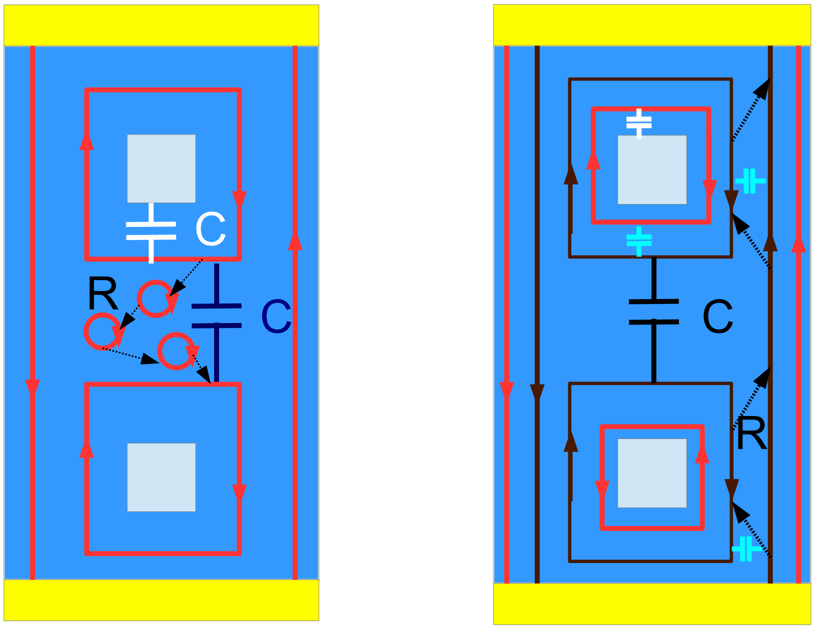

Supplementary Figure 8: The edge state distributions and capacitances within the SP theories. Sketches present edge states considering different average filling factors, $\nu = 2$ (on the left) and $\nu = 4$ (on the right), where also the capacitances between the edge states and contacts are presented by inner (white) symbols. Similar to Halperin's discussion of a Corbino geometry, the capacitance between contacts are determined by the bulk incompressible region, however, here the excitation is perpendicularly imposed with respect to edge states. Interestingly, even at the transition between $\nu = 4$ to $\nu = 2$ plateau the inner edge states should provide a high capacitance. Namely one can expect scattering between $\nu = 4$ edge states, which would yield a huge impedance even at the transition intervals

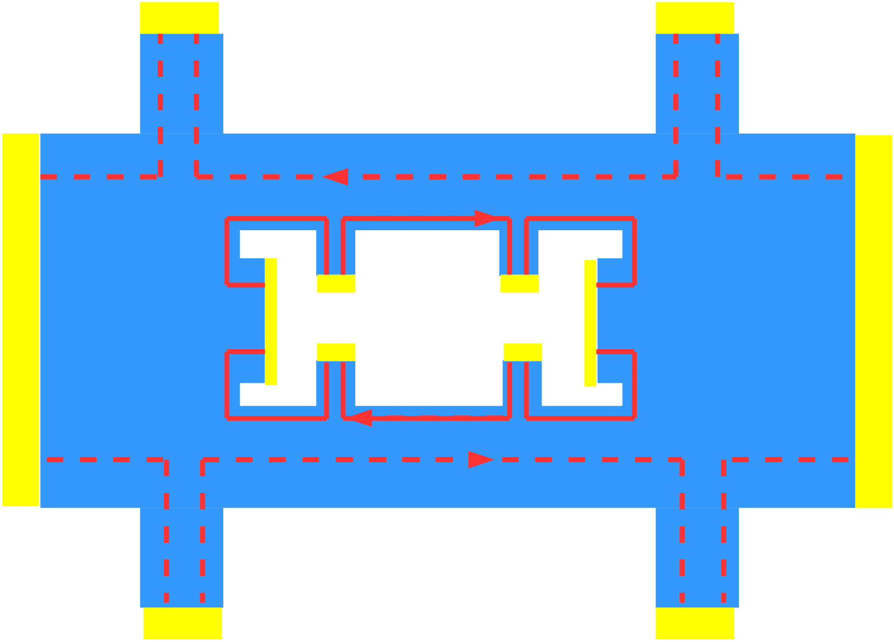

Supplementary Figure 9: Sketch of an anti-Hall bar. Once an anti-Hall bar is “embedded” into a Hall bar, one expects to have two independent edge state configurations which are decoupled by the incompressible region in between. Here it is essential to realise that, the edge states of the anti-Hall bar connect the source and drain contacts and do not encircle entirely any of the contacts.

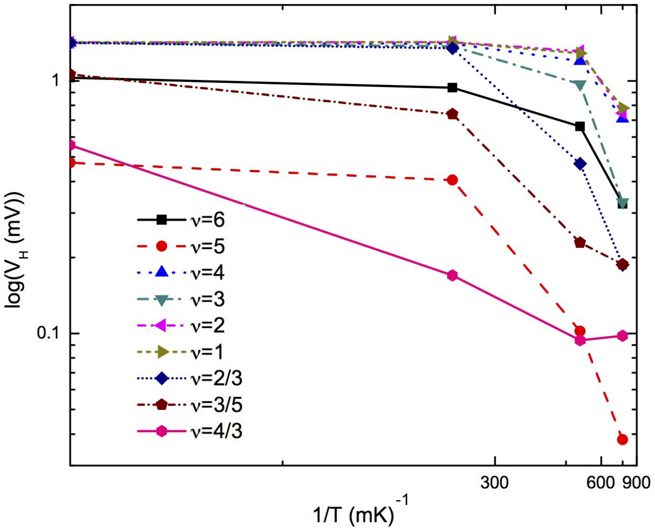

Supplementary Figure 10: The Arrhenius plot of Max(V_{AB}). The maximum of the voltage drop between contacts A and B considering different filling factors. One can see that, the activated behaviour of the well developed integer states coincide nicely, whereas for fractional states the gap value is scattered depending on the filling factor.

Supplementary References

[1] D. Weiss et .al, "Density of states in Landau level tails of GaAs-AlGaAs-heterostructures," Springer Series in Solid-State Sciences, vol. 67, p. 204, 1986.

[2] D. J. Thouless, M. Kohmoto, M. P. Nightingale, and M. den Nijs, "Quantized hall conductance in a two-dimensional periodic potential," Phys. Rev. Lett., vol. 49, p. 405, 1982.

[3] R. B. Laughlin, Phys. Rev. B, vol. 23, p. 5632, 1981.

[4] M. Z. Hasan and C. L. Kane, "Colloquium: Topological insulators," Reviews of Modern Physics, vol. 82, pp. 3045–3067, Oct. 2010.

[5] B. I. Halperin, "Self-consistent local-equilibrium model for density profile and distribution of dissipative currents in a hall bar under strong magnetic fields," Phys. Rev. B, vol. 25, p. 2185, 1982.

[6] M. Büttiker, "Four-terminal phase-coherent conductance," Phys. Rev. Lett., vol. 57, p. 1761, 1986.

[7] A. K. Evans, L. I. Glazman, and B. I. Shklovskii, "Coulomb blockade in the quantum-Hall-effect state," Phys. Rev. B, vol. 48, pp. 11120–11127, Oct. 1993.

[8] R. G. Mani, "Experimental technique for realizing dual and multiple Hall effects in a single specimen," EPL (Europhysics Letters), vol. 34, pp. 139–144, Apr. 1996.

[9] C. Uiberacker, C. Stecher, and J. Oswald, "Microscopic details of the integer quantum Hall effect in an anti-Hall bar," Phys. Rev. B , vol. 86, p. 045304, July 2012.
